# A difficult case of Hodgkin lymphoma mimicking tuberculosis in a young female patient: A case report

**DOI:** 10.1002/ccr3.7290

**Published:** 2023-05-01

**Authors:** Bakri Roumi Jamal, Mohamad Ali Farho, Mohamad Moafak Hariri, Abdullah Khoury

**Affiliations:** ^1^ Faculty of Medicine University of Aleppo Aleppo Syria; ^2^ CME Office, Faculty of Medicine University of Aleppo Aleppo Syria; ^3^ Department of Pulmonology, Faculty of Medicine, Aleppo University Hospital University of Aleppo Aleppo Syria

**Keywords:** B‐cell lymphoma, case report, cavitation, Hodgkin lymphoma, lymphadenopathy, misdiagnose, tuberculosis

## Abstract

Due to overlapping clinical symptoms, it may be difficult to distinguish between Hodgkin lymphoma and tuberculosis. Repeated investigations, especially biopsy and histopathological examination, may be helpful to establish a correct diagnosis. Herein, we present a case of Hodgkin's lymphoma misdiagnosed as tuberculosis so we always need evidence‐based medicine in our medical life.

**Abstract:**

Distinguishing between Hodgkin's Lymphoma and tuberculosis is challenging. A 23‐year‐old patient has been diagnosed with tuberculosis; based on clinical and radiological findings. After the therapy, her condition worsened. Cervical lymphadenopathy was detected. A biopsy was performed and the diagnosis was classical Hodgkin lymphoma. After the treatment, the patient improved significantly.

## 1 INTRODUCTION

Hodgkin lymphoma (HL) is a B‐cell lymphoma, representing 1% of all latest diagnosed tumors worldwide and 15% of lymphomas [85% of these are non‐Hodgkin lymphoma (NHL)].^1^ According to the last global data analysis, the incidence of HL is 0.98 per 100,000 people and is still on the rise, particularly among females, Asians, and young people.^2^ The most common manifestation is asymptomatic lymphadenopathy in cervical, and supraclavicular.^3^ Mediastinal lymph nodes are the most common regions where lymphoma appears and mediastinum enlargement is the most prominent X‐ray manifestation.^3^ Moreover, it is difficult to diagnose the involvement of lymphoma in the lungs. Mass or mass‐like consolidation larger than 1 cm is the most common CT finding of lymphoma.^4^ In addition, cavitation in the lungs caused by lymphoma is a rare manifestation.^5^ However, it could be challenging to distinguish between HL and tuberculosis (TB) due to the overlap of clinical symptoms leading to misdiagnosis.^6^ Fever, night sweats, and weight loss are symptoms of both TB and HL.^7^ In the present case report, we report a difficult rare case of a female patient with HL mimicking TB because of the similarity of clinical and radiological findings.

## 2 CASE PRESENTATION

**FIGURE 1:**
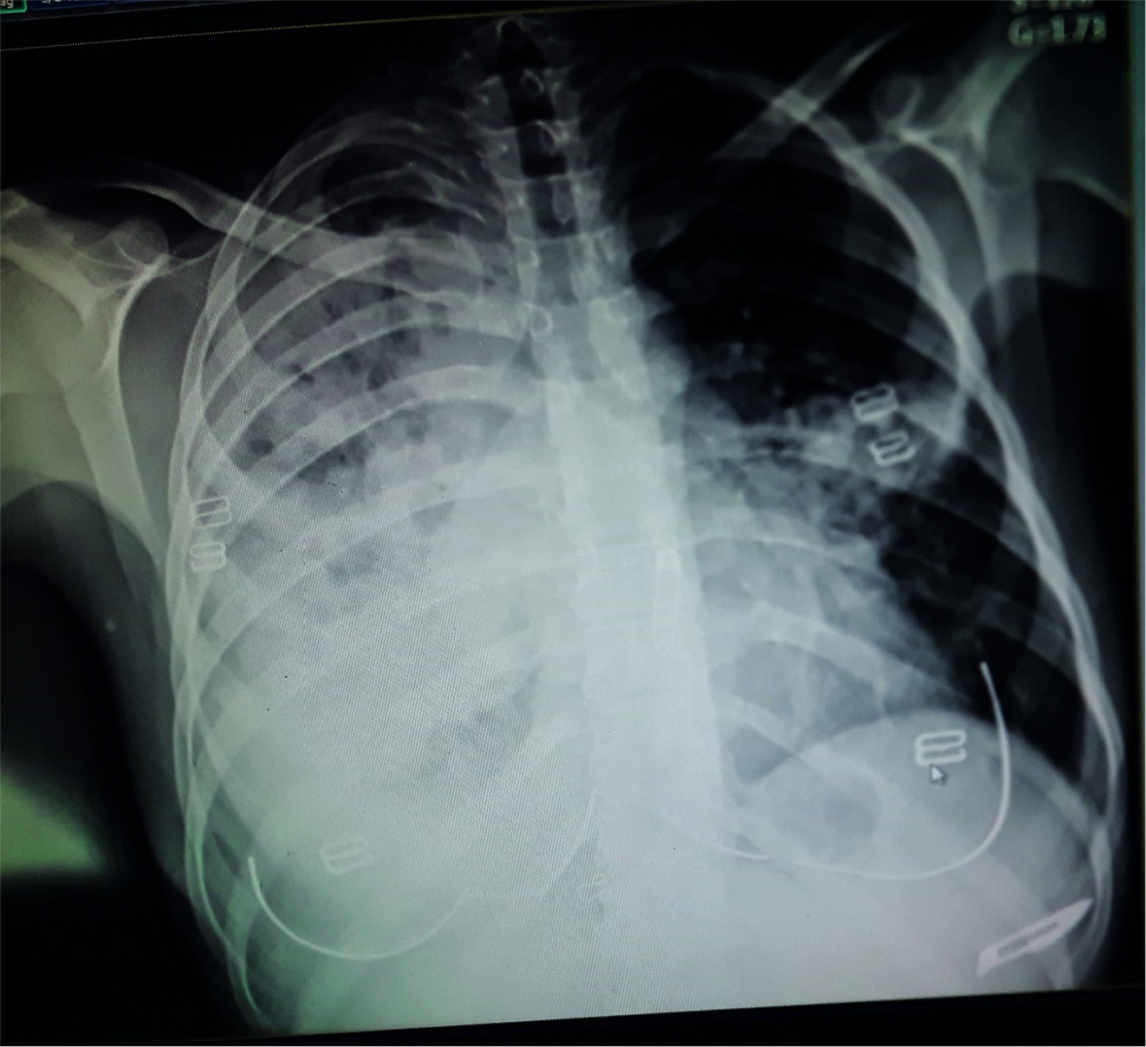
Chest x‐ray (CXR) demonstrated density, infiltrates, and cavities in the lungs.

**FIGURE 2:**
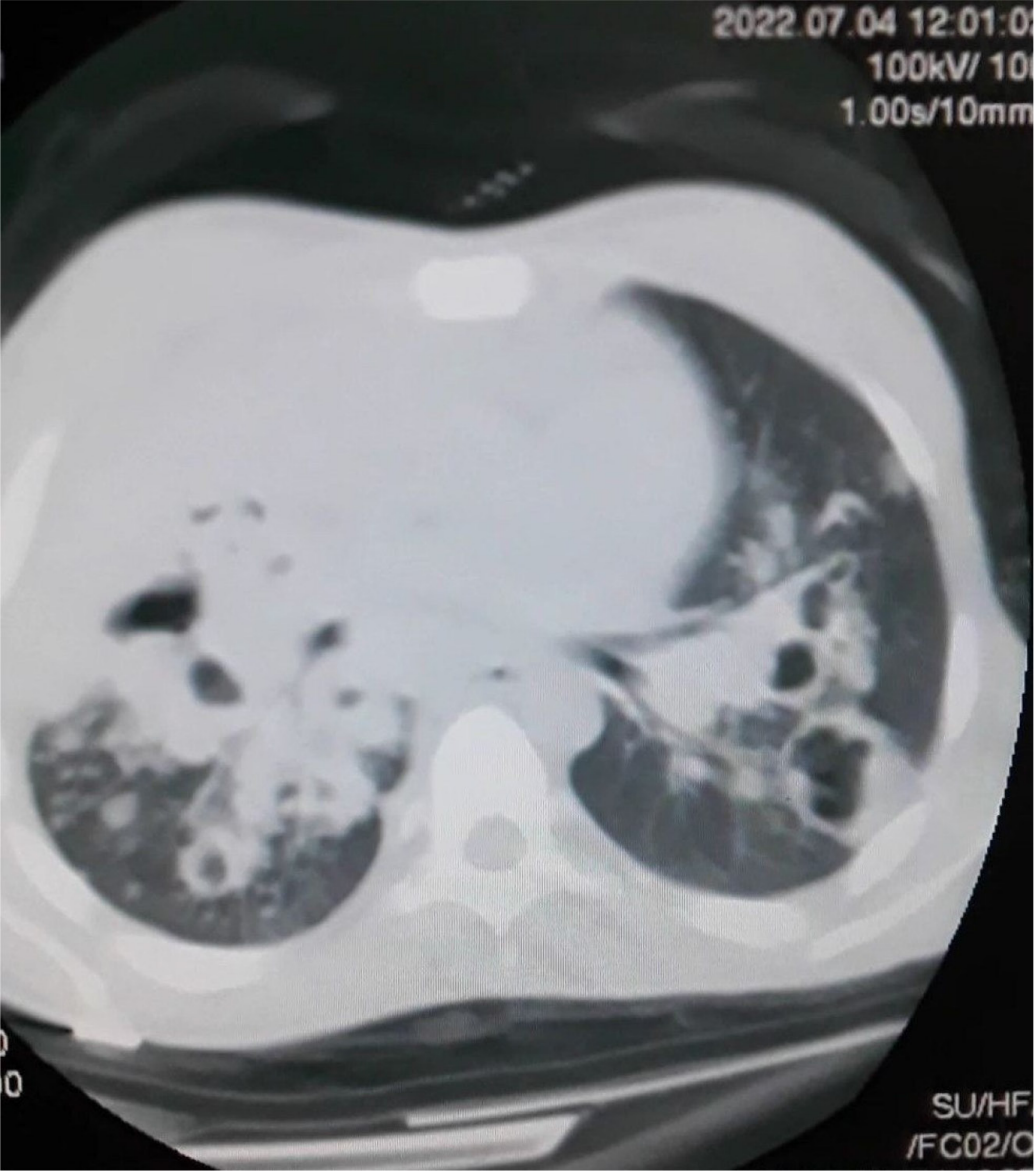
Computed tomography (CT) scan demonstrated density, infiltrates, and cavities in the lungs.

**FIGURE 3:**
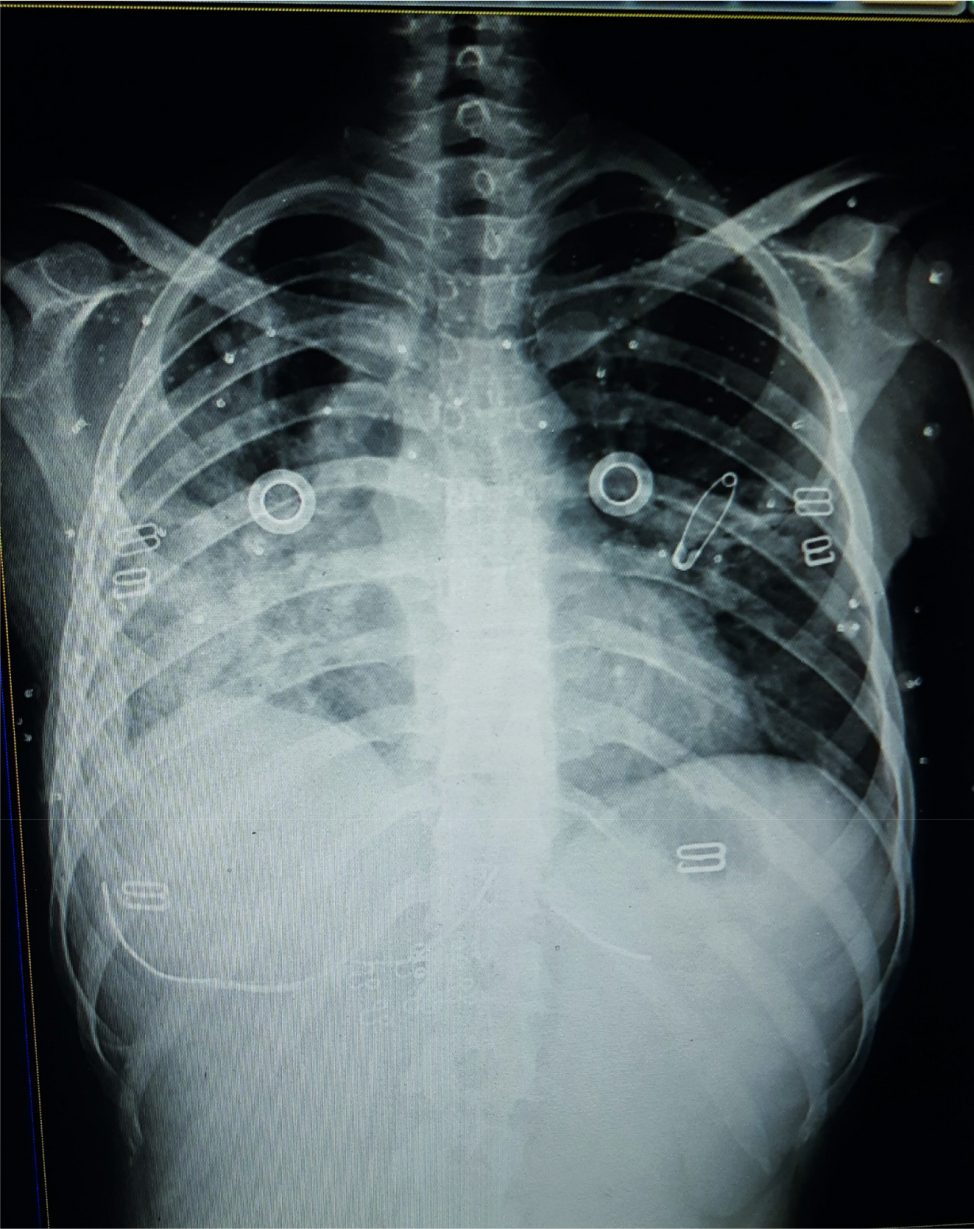
The second chest x‐ray CXR) image showed an improvement.

A 23‐year‐old female patient was admitted to the hospital with complaints of dyspnea associated with severe chest pain, productive cough, and dizziness for 3 days. She also complained of generalized weakness, loss of appetite, and weight loss. She was a non‐smoker, and non‐alcoholic with no significant medical or surgical history. She has a family history of TB. There were no palpable peripheral lymph nodes present anywhere in the body. Laboratory findings showed mild anemia with elevated C‐reactive protein (CRP). Chest X‐ray (CXR) and computed tomography (CT) demonstrated density, infiltrates, and cavities in the lungs (Figures 1 and 2). Based on clinical and radiological findings, the patient has been diagnosed with TB although the sputum culture result was negative for TB and she received quadruple therapy (isoniazid, rifampin, pyrazinamide, ethambutol). After 2 months, she has again hospitalized with general weakness and prominent cervical lymphadenopathy during the physical examination. A biopsy was performed and immunostains for CD15, CD30, and CD20 markers were positive, and the CD3 marker stains background cells. This panel is consistent with classical HL and the patient was referred to the oncology center for further treatment. The patient was treated with the chemotherapy ABVD regimen (bleomycin, doxorubicin hydrochloride, vinblastine, and dacarbazine). After one course of treatment, the patient showed a significant improvement, with no cervical lymph node enlargement. Moreover, we performed another CXR for the patient, and there has been improvement (Figure 3). The patient has been placed on a follow‐up program to ensure she is in a good health.

## 3 DISCUSSION

Lymphoma is a type of hematological malignancy that is usually found in the lymph nodes or the lymphatic system. The two main types of lymphoma are NHL (~85% of cases) and HL (~15% of cases).^1^

Hodgkin lymphoma (HL) is a B‐cell lymphoma, potentially curable with distinct histology and clinical features. It is characterized by few malignant cells and numerous immune effector cells in the tumor microenvironment. Features of HL include unexplained weight loss, fever, night sweats, chest pain, cough, and pain at sites of disease.^2^

Pulmonary lesions occur in advanced stages of HL but primary pulmonary involvement is very rare and unlikely. In addition, cavitation in the lungs caused by lymphoma is a rare manifestation.^4^

Tuberculosis (TB) is a multisystemic disease caused by mycobacterium tuberculosis that presents countless different symptoms and manifestations. Pulmonary TB clinical features usually are cough, weight loss, fever, night sweats, hemoptysis, and chest pain. TB shares clinical manifestations with many diseases including HL, where one can mimic the other, making their diagnosis a challenge and leading to a misdiagnosis.^1^, ^8^

The relationship between HL and TB has been proven but it is difficult to distinguish due to several points; the first is that TB and HL present common B symptoms. Furthermore, the lesions may originate at sites where performing a biopsy is difficult.^9^ Our study has a double interest; it shows pulmonary involvement at the early stage of HL, and the most important one is to reveal the diagnostic difficulty between HL and TB, especially when an initial radiological examination reveals a cavity lesion. In this case, we will discuss several basic points that were discussed to reach the final diagnosis, including the differential diagnosis of tuberculosis and lymphoma, in addition to their association. At the first presentation, the symptoms of dyspnea, chest pain, productive cough, dizziness, generalized weakness, loss of appetite, and weight loss were unspecific to a particular disease. The first CXR demonstrated cavitary pulmonary lesions and pleural effusion. Based on the symptoms, family history, and radiological images, the initial diagnosis was in favor of TB and the treatment began since the most common TB site is in the lung and its main feature on the X‐Ray chest image is cavitary lesions.^1^ The points that support the clinical diagnosis of tuberculosis in our patient are clinical symptoms and radiological images that showed tuberculous cavities in the lung, her family history of tuberculosis, the country is endemic to tuberculosis and no studies are showing actual prevalence in the community, and lymphadenopathy was not present initially. After 2 months of quadruple TB treatment (isoniazid, rifampin, pyrazinamide, ethambutol); the clinical symptoms and radiological images continued to worsen and the sputum culture result was negative for TB so it is either multidrug‐resistant TB or it is not TB. The reason of continuing the treatment of tuberculosis for 2 months was based on all the points that support this diagnosis, and the patient did not go to the hospital during this 2 months duration because she lived in a remote rural area and until a new symptom appeared, which was lymphadenopathy. The patient developed an enlarged cervical lymph node. A biopsy was performed and the immunostains for CD15, CD30, and CD20 were positive. As a result, the diagnosis was classical HL and the chemotherapy treatment began.^5^

After the chemotherapy courses, the symptoms and the pulmonary lesions improved, so we are completely denying the diagnosis of TB since chemotherapy has enhanced the TB. In the present case, the reasons behind the delay in diagnosis can be summarized as follows. (i) The patient was a middle‐aged woman who presented with cavitary pulmonary lesions, raising the suspicion of TB. (ii) Primary pulmonary involvement of Hl is very rare.

## 4 CONCLUSION

Our report suggests for clinicians to seriously discuss the diagnosis of TB, especially when anti‐TB treatment failed. Repeated investigations, especially biopsy and histopathological examination, may be helpful to establish a correct diagnosis.

## AUTHOR CONTRIBUTIONS

**Bakri Roumi Jamal:** Project administration; writing – original draft. **Mohamad Ali Farho:** Project administration; writing – original draft. **Mohamad Moafak Hariri:** Writing – original draft. **Abdullah Khoury:** Supervision.

## FUNDING INFORMATION

The authors declare no source of funding for this manuscript from any organization or any institution.

## CONFLICT OF INTEREST STATEMENT

The authors want to declare that none of them is or was employed by any government agency that has any function other than research and education, and none of them is submitting this manuscript as an official representative or on behalf of the government.

## CONSENT

Written informed consent was obtained from the patient to publish this report based on the journal's patient consent policy.

## Data Availability

Data sharing is not applicable to this article as no new data were created or analyzed in this study.
